# Characterization of a Deep Sea *Bacillus toyonensis* Isolate: Genomic and Pathogenic Features

**DOI:** 10.3389/fcimb.2021.629116

**Published:** 2021-03-10

**Authors:** Jing-chang Luo, Hao Long, Jian Zhang, Yan Zhao, Li Sun

**Affiliations:** ^1^ CAS and Shandong Province Key Laboratory of Experimental Marine Biology, Institute of Oceanology, Center for Ocean Mega-Science, Chinese Academy of Sciences, Qingdao, China; ^2^ Laboratory for Marine Biology and Biotechnology, Pilot National Laboratory for Marine Science and Technology, Qingdao, China; ^3^ College of Earth and Planetary Sciences, University of Chinese Academy of Sciences, Beijing, China; ^4^ State Key Laboratory of Marine Resource Utilization in South China Sea, Hainan University, Haikou, China; ^5^ School of Ocean, Yan Tai University, Yantai, China

**Keywords:** *Bacillus toyonensis*, deep-sea hydrothermal vent, virulence, infection, genome

## Abstract

*Bacillus toyonensis* is a group of Gram-positive bacteria belonging to the *Bacillus cereus* group and used in some cases as probiotics or biocontrol agents. To our knowledge, *B. toyonensis* from the deep sea (depth >1,000 m) has not been documented. Here, we report the isolation and characterization of a *B. toyonensis* strain, P18, from a deep sea hydrothermal field. P18 is aerobic, motile, and able to grow at low temperatures (4°C) and high concentrations of NaCl (8%). P18 possesses a circular chromosome of 5,250,895 bp and a plasmid of 536,892 bp, which encode 5,380 and 523 genes, respectively. Of these genes, 2,229 encode hypothetical proteins that could not be annotated based on the COG database. Comparative genomic analysis showed that P18 is most closely related to the type strain of *B. toyonensis*, BCT-7112^T^. Compared to BCT-7112^T^, P18 contains 1,401 unique genes, 441 of which were classified into 20 COG functional categories, and the remaining 960 genes could not be annotated. A total of 319 putative virulence genes were identified in P18, including toxin-related genes, and 24 of these genes are absent in BCT-7112^T^. P18 exerted strong cytopathic effects on fish and mammalian cells that led to rapid cell death. When inoculated *via* injection into fish and mice, P18 rapidly disseminated in host tissues and induced acute infection and mortality. Histopathology revealed varying degrees of tissue lesions in the infected animals. Furthermore, P18 could survive in fish and mouse sera and possessed hemolytic activity. Taken together, these results provide the first evidence that virulent *B. toyonensis* exists in deep sea environments.

## Introduction

Deep hydrothermal vents are localized areas of the deep sea with high tectonic activities that cause the escaping of hot, mineral-rich water from the sea floor, which forms chimney-like black or white smokers. Compared to other areas of the deep ocean, hydrothermal regions are rich in various sulfur compounds and have large amounts of small molecules of hydrocarbons, which provide the basis for the development of chemoautotroph microbial communities that can utilize the sulfur compounds and hydrocarbons (Marteinsson et al., 1996; Ruger et al., 2000; Burgaud et al., 2009; Le Calvez et al., 2009; Wang et al., 2020). The food and energy produced by the microorganisms enable the growth of invertebrate animals, such as mussels, tube worms, and crabs, thus forming the deep-sea food web (Dover, 2002; Nishijima et al., 2010; Van Dover, 2014).

*Bacillus* spp. are present in soil, air, and the deep-sea (Liu et al., 2006; Stenfors Arnesen et al., 2008). In animals, *B. cereus* can be a transient presence in the guts of mammals, and for some insects, *B. cereus* is part of the gut microbial communities (Jensen et al., 2003). They have been studied mostly as probiotics for animal feeds and medicines (Cutting, 2011; Elshaghabee et al., 2017). However, in the *B. cereus* group, which is a species complex within the *Bacillus* genus, some members, such as *Bacillus anthracis*, *Bacillus thuringiensis*, and *B. cereus*, are well known as pathogens and can cause fatal diseases in humans and animals (Bottone, 2010). *Bacillus anthracis* causes anthrax mediated by two key virulence factors: a tripartite toxin and a poly-D-glutamic acid capsule (Bourgogne et al., 2003; Liu et al., 2014). *Bacillus thuringiensis*, which has been used in agriculture as a biopesticide, produces proteins that aggregate into crystals and are toxic to insects (Berry et al., 2002; Bravo et al., 2007; Li et al., 2008). Some members of the *Bacillus cereus* group can produce several toxins, including the single-protein toxin, cytotoxin K (CytK), and the two protein toxin complexes formed by hemolysin BL (Hbl) and the non-hemolytic enterotoxin (Nhe) (Stenfors Arnesen et al., 2008; Ehling-Schulz et al., 2019).

*Bacillus toyonensis* is a member of the *Bacillus cereus* group, with strain BCT-7112^T^ being the type strain (Jimenez et al., 2013). Strain BCT-7112^T^ is a soil isolate and has been used for many years as an additive of animal feeds. In a recent study, it was demonstrated that although BCT-7112^T^ possesses the genes coding for hemolysin BL (Hbl) and the non-hemolytic enterotoxin (Nhe), the expression levels of these genes were undetectable or very low compared to the *B. cereus* reference strains, suggesting that the ability of BCT-7112^T^ to produce functional enterotoxins is low or unlikely (Abdulmawjood et al., 2019). Recently, two other members of *B. toyonensis* were reported as potentially novel agents for biocontrol of plants (Lopes et al., 2017; Rojas-Solis et al., 2020). *B. toyonensis*-like strains have been shown to exist in neritic regions (Okaiyeto et al., 2015; Tallur et al., 2016), however, to our knowledge, no *B. toyonensis* isolates from the deep sea (depth >1,000 m) have been documented.

In this study, we reported the isolation of a *B. toyonensis* strain, P18, from a deep sea hydrothermal field and the analysis of its genomic, biological, and virulence features. We found that P18 possesses unique genetic traits and was able to cause lethal infection in fish and mice upon artificial inoculation. Our results provide the first evidence that pathogenic *B. toyonensis* exits in the deep-sea environment.

## Materials and Methods

### Ethics Statement

The experiments involving live animals were approved by the Animal Ethics Committee of Institute of Oceanology, Chinese Academy of Sciences. All methods were carried out in accordance with the relevant guidelines.

### Animals

The mice and fish used in this study were purchased and maintained as reported previously (Gu et al., 2019). Briefly, clinically healthy BALB/c mice (female, 8–10 weeks, and 14 ± 2 g) were purchased from Qingdao Daren Fortune Animal Technology Co., Ltd. Clinically healthy Japanese flounder (*Paralichthys olivaceus*) (15 ± 0.5g) were purchased from a local fish farm in Shandong Province, China and maintained at 20°C in aerated seawater in the laboratory. For tissue collection, the fish and mice were euthanized with an overdose of tricaine methanesulfonate (Sigma, St. Louis, USA) and ketamine (80 mg/kg) (Ketavet, Pfizer, Berlin, Germany), respectively (Gu et al., 2019).

### Cell Culture

The cell lines used in this study were cultured as reported previously (Gu et al., 2019; Zhao et al., 2019). Briefly, RAW264.7 cells and Hela cells were cultured in DMEM (Gibco, USA) with 10% fetal bovine serum (Gibco, USA) and 1% penicillin and streptomycin (Beyotime Biotechnology, China) at 37°C and 5% CO2. FG-9307 cells were cultured in L-15 (Genom, China) with 10% fetal bovine serum and 1% penicillin and streptomycin at 24°C.

### Bacterial Isolation

P18 was isolated from a sediment sample collected from the hydrothermal field in southern Okinawa Trough (127°04′30.980″ E, 27°16′01.064″ N, depth of 1446 m), where the water temperature was around 4°C. The sediment sample was obtained by push-cores on the Remotely Operated Vehicle (ROV) equipped on the KEXUE vessel (Sun et al., 2015). The sample was plated on Marine agar 2216 medium (Sun et al., 2015) and incubated at 28°C under aerobic conditions for 3 days. One of the colonies that appeared on the plate was named P18, which, after re-purification, was stored at −80°C with 30% (v/v) glycerol.

### Morphology and Growth of P18

For morphological observation, P18 was cultured in Marine 2216E medium at 28°C to an OD_600_ of 0.8. The culture was centrifuged at 5,000 rpm for 10 min, and the bacterial pellet was re-suspended in ddH_2_O. For spore observation, P18 was cultured at 28°C in Marine 2216E medium containing 5mg/L MnSO_4_ for 3 days with shaking (180 rpm/min) (Osadchaia et al., 1997); the culture was then centrifuged and re-suspended in ddH_2_O. After fixation with glutaraldehyde and dehydration with acetone, the vegetative cells and spores of P18 were observed with a transmission electron microscope (Hitachi, JEM-2100, Japan). The spores were also stained with a Spore Staining Kit (HaiBo, China) and observed with a light microscope (Ti-S/L100, Nikon, Japan). The temperature range of growth was determined by culturing P18 at 4, 16, 28, 37, and 50°C in Marine 2216E medium for 36 h to 7 days. The pH range was determined by culturing P18 in Marine 2216E medium of different pH (pH 5–10, with an increment of 1 pH unit). The NaCl range was determined by culturing P18 in 2216E medium with different salinities (NaCl 0–8%, w/v, with an increment of 1%). The motility of P18 was determined as reported previously (Gu et al., 2019).

### Genome Analysis

Genome sequencing was carried out by Novogene (Beijing, China) as reported previously (Zhao et al., 2019). Briefly, clean data were obtained by controlling the quality of the original data and filtering out the low-quality sequences for subsequent analysis. SMRT software (Link V5.0.1) (Berlin et al., 2015) was used to assemble reads. Plasmid sequences were distinguished from chromosomal sequence based on the method of Gomez-Escribano et al. (2015). Coding genes were predicted with GeneMarkS 4.28 software (http://topaz.gatech.edu/) (Besemer et al., 2001). Genomic islands were predicted using IslandPath-DIOMB 0.2 software (Hsiao et al., 2003). ncRNA sequences were predicted using rRNAmmer 1.2 (Lagesen et al., 2007) and tRNAscan 1.3.1 (Schattner et al., 2005) softwares and the Rfam (Griffiths-Jones et al., 2005) database. Repeated sequences and prophages were predicted using RepeatMasker 4.0.5 (Chen, 2004) and phiSpy 2.3 (Akhter et al., 2012) softwares, respectively. All programs were run with default settings. Functional annotation was performed using the following databases: KEGG (http://www.genome.jp/kegg/) (Kanehisa et al., 2004), COG (http://www.ncbi.nlm.nih.gov/COG/) (Tatusov et al., 2003), SwissProt (http://www.uniprot.org/) (Bairoch and Apweiler, 2000), GO (http://www.geneontology.org/) (Ashburner et al., 2000), and NR (http://www.ncbi.nlm.nih.gov/RefSeq/) (Li et al., 2002). Diamond (v0.7.9.58) blastp was used for the analysis with the COG, KEGG, and NR databases (identity >40% and E-value <1E-05). Blast was used for the analysis with the SwissProt database (identity >40% and E-value <1E-05). Gene mapping was performed using Circos software by combining the assembled genome sequence with the predicted results of the coding genes. Virulence related genes were predicted using the VFDB (Virulence Factors of Pathogenic Bacteria Database; http://www.mgc.ac.cn/VFs/) database (Chen et al., 2005) with Diamond (v0.7.9.58) blastp (identity >40% and E-value <1E-05). The genomes of P18 and BCT-7112^T^ (Harris, 2007) were compared using MUMmer 3.23 software (Delcher et al., 2003) to determine the collinearity. LASTZ 1.02.00 software was used to confirm Translocation/Trans, Inversion/Inv, and Trans+Inv regions between P18 and BCT-7112^T^. Gene clusters were identified using the cd-hit software (Li and Godzik, 2006), with identity >50% and length difference <30%. The average nucleotide identity (ANI) between P18 and other strains of *Bacillus* (**Supplementary Table S1**) was calculated using the EzBiocloud web service (https://www.ezbiocloud.net/tools/ani) (Kim et al., 2014). The chromosome and plasmid sequences of P18 have been deposited in GenBank under the accession numbers CP064875 and CP064876, respectively.

### Infection Assay

*In vivo* infection was performed as reported previously (Gu et al., 2019). Briefly, for infection of flounder, the fish were injected intramuscularly with P18 at the dose of 3 × 10^4^ to 3 × 10^6^ CFU/g (with 10-fold interval) and monitored for mortality for 10 days. For the comparison of the mortality-inducing capacity of P18 and *Bacillus subtilis* 168 [purchased from China General Microbiological Culture Collection Center (CGMCC, http://www.cgmcc.net)], the bacteria were cultured to an OD_600_ of 0.8. The bacteria were collected by centrifugation and resuspended in PBS. The same dose of P18 and *B. subtilis* 168 were used to inject flounder as above. For infection of mice, BALB/c mice were injected intraperitoneally with P18 at the dose of 6.25 × 10^5^ to 5 × 10^6^ CFU/g (with 2-fold interval) and monitored for mortality for 10 days. For all infections, the control groups were injected with PBS. The median lethal dose (LD50) was determined as reported previously (Gu et al., 2019). For the detection of bacterial dissemination in host tissues, liver, spleen, kidney, and blood of flounder (five fish/time point) were sampled at 12, 24, and 48 hpi; liver, spleen, kidney, and intestine of mice (three mice/time point) were sampled at 2, 4, and 6 hpi. The tissues were homogenized in PBS, and bacterial numbers in the homogenates were determined by plate count as reported previously (Zhao et al., 2019).

### Histopathology

For histopathological analysis, the tissues prepared above were immersed in 4% PFA and stored at room temperature. Tissue sectioning and HE staining were performed by Wuhan Servicebio Technology Co., Ltd. (Wuhan, China). After dewaxing, hematoxylin staining, eosin staining, and dehydration, the tissue sections were observed with an optical microscope (Eclipse E100, Nikon, Japan).

### Cellular Infection

P18 was cultured in L-15 or DMEM medium at 28°C overnight. The culture was centrifuged at 8,000 rpm, and the bacteria and the supernatant were collected. The bacteria were resuspended in L-15 or DMEM. The supernatant was filtered with a 0.22 Millipore filters (Millex^®^-GP, Cork, Ireland). FG-9307, RAW264.7, and Hela cells were culture as described above for overnight in 24-well plates. The cells were infected with P18 at a MOI of 3:1 or treated with the bacterial supernatant (5%, v/v). After incubation at 28°C for 0.5–1.5 h, the cells were observed with a light microscope.

### Serum Resistance and Hemolytic Activity

Serum resistance and hemolytic activity were analyzed as reported previously (Gu et al., 2019). Briefly, to examine the hemolytic activity of P18, P18 and *B. subtilis* 168 were cultured to an OD_600_ of 0.8. The bacteria were collected by centrifugation and resuspended in PBS. Ten microliters of the bacterial suspension were added to the filter paper on a LB agar plate containing 5% sterile sheep blood (Hopebio, China). Triton X-100 and PBS were used as the positive and negative controls, respectively. The plate was incubated at 28°C for 24 h and then observed for hemolysis.

## Results

### Morphological and Growth Characteristics of P18

**Figure 1:**
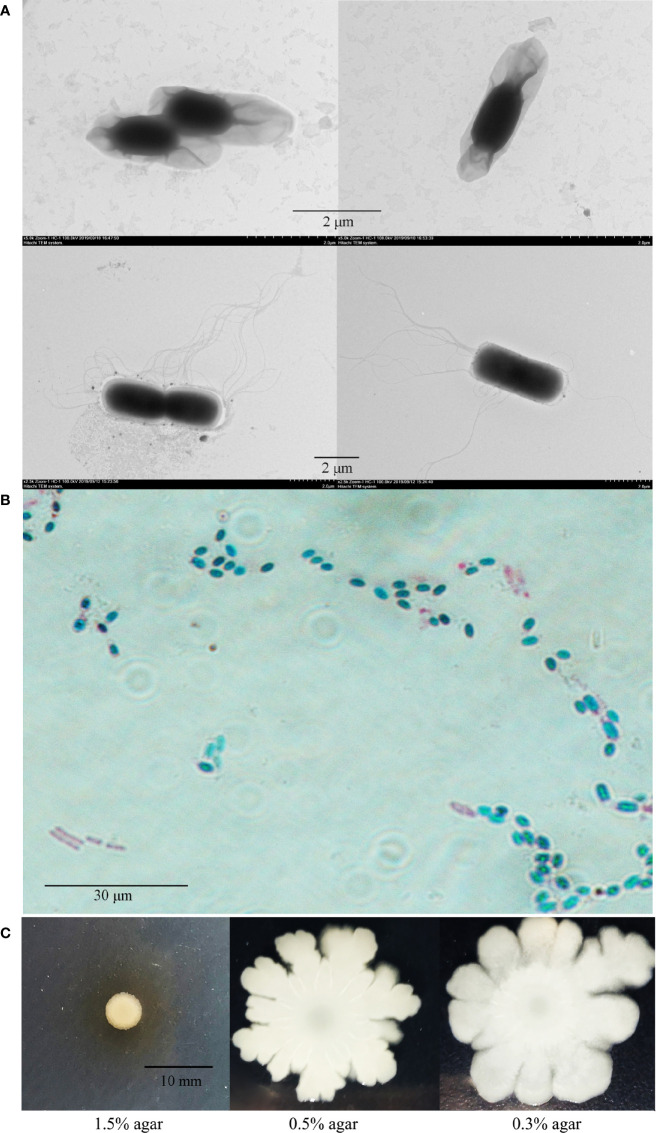
Morphology and motility of P18. **(A)** P18 cells of spore (upper) and vegetative (lower) growth observed with a transmission electron microscope. **(B)** P18 cells in vegetative (pinkish red) and spore (bluish green) forms observed with a microscope. **(C)** Growth of P18 in marine 2216E containing 0.3, 0.5, or 1.5% (w/v) agar.

Strain P18 was isolated from a deep-sea sediment sample collected from the hydrothermal field in southern Okinawa Trough (127°04′30.980″ E, 27°16′01.064″ N, depth 1446 m). Transmission electron microscopy (TEM) showed that P18 was rod-shaped and exhibited peritrichous flagella (**Figure 1A**). It is able to produce endospores under specific conditions (**Figure 1B**). P18 exhibited swimming and swarming abilities in 0.3 and 0.5% agar, respectively (**Figure 1C**). P18 grew well at pH 5–9, 4–37°C, and in the presence of up to 8.0% NaCl (**Supplementary Figure S1**).

### Genome Features of P18

**Figure 2:**
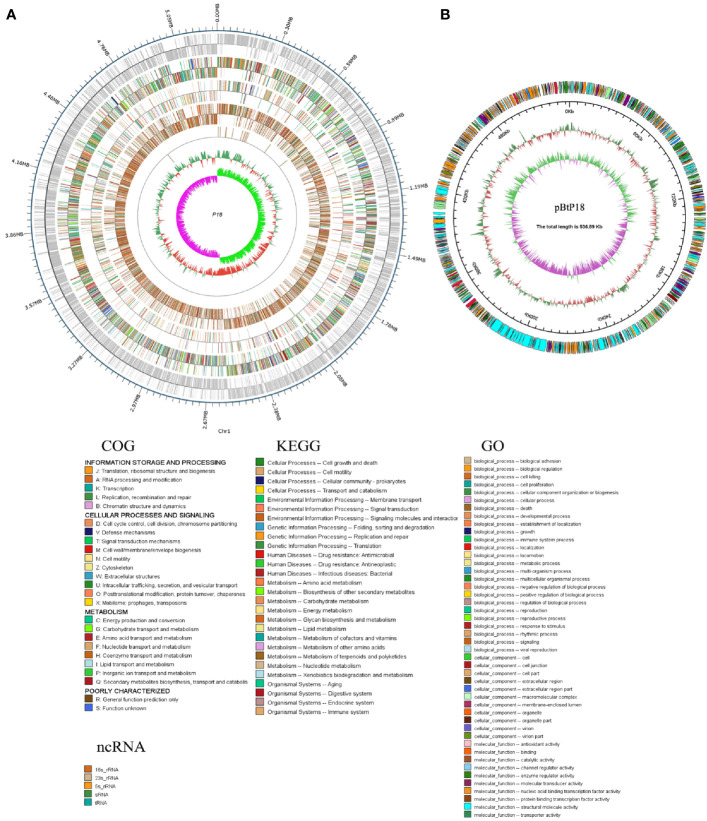
Genomic maps of the P18 chromosome **(A)** and plasmid **(B)**. **(A)** From the outside circle to the inside circle are the coordinates of the genome sequence, annotation information of gene function (based on COG, KEGG, and GO databases), ncRNA, GC content of the genome, distribution of the value of GC skew. **(B)** From the outside circle to the inside circle are COG annotation information, the coordinates of the plasmid sequence, GC content of the plasmid, and distribution of the value of GC skew.

P18 contains a circular chromosome of 5 250 895 bp and a plasmid (named pBtP18) of 536 892 bp, with an average G+C content of 35.42 and 32.86%, respectively (**Figure 2**; **Table 1**). The chromosome and the plasmid encode 5,380 and 523 genes, respectively, with average gene lengths of 820 and 754 bp, respectively. There are 2,229 genes of P18 that encode hypothetical proteins and could not be annotated functionally based on the COG database. P18 has 14 sets of rRNAs (5S, 16S, and 23S rRNA), 105 tRNAs, and 4 sRNAs, all of which are present on the chromosome, and 20 genomic islands (GIs), 16 of which are present on the chromosome (**Table 1**). In addition, P18 possesses 260 interspersed repeats, 367 tandem repeats, 298 mini-satellites, and 1 microsatellite. P18 exhibits 98.67, 91.04, 91.22, 91.43, and 91.54% ANI values with *Bacillus toyonensis* BCT-7112^T^, *Bacillus luti* TD41^T^, *Bacillus wiedmanni*i FSL W8-0169^T^, *Bacillus cereus* ATCC 14579^T^, and *Bacillus thringiensis* ATCC 10792^T^, respectively (**Supplementary Table S1**). Based on these results, P18 was classified as a member of *B. toyonensis*.

**Table 1:** Features of P18 and BCT-7112^T^.

Feature	P18			BCT-7112^T^			
	Chromosome	Plasmid	Total	Chromosome	Plasmid 1	Plasmid 2	Total
Size (bp)	5,250,895	536,892	5,787,787	4,940,474	76,974	7,971	5,025,419
Number of genes	5,380	523	5,903	5,131	97	11	5,239
Coding region (%)	84.02	73.45	82.92	84.33	75.48	60.58	84.13
Average gene length (bp)	820	754	813	812	599	439	807
GC content (%)	35.42	32.86	35.18	35.6	32.93	31.4	35.55
tRNA	105	0	105	97	0	0	97
rRNA	14 (5s-16s-23s)	0	14 (5s-16s-23s)	12 (5s-16s-23s)	0	0	12 (5s-16s-23s)
sRNA	4	0	4	6	1	0	7
Genomic islands	16	4	20	16	1	0	17

### Comparative Analysis of the Genomes of P18 and Its Close Homologue BCT-7112^T^

**Figure 3:**
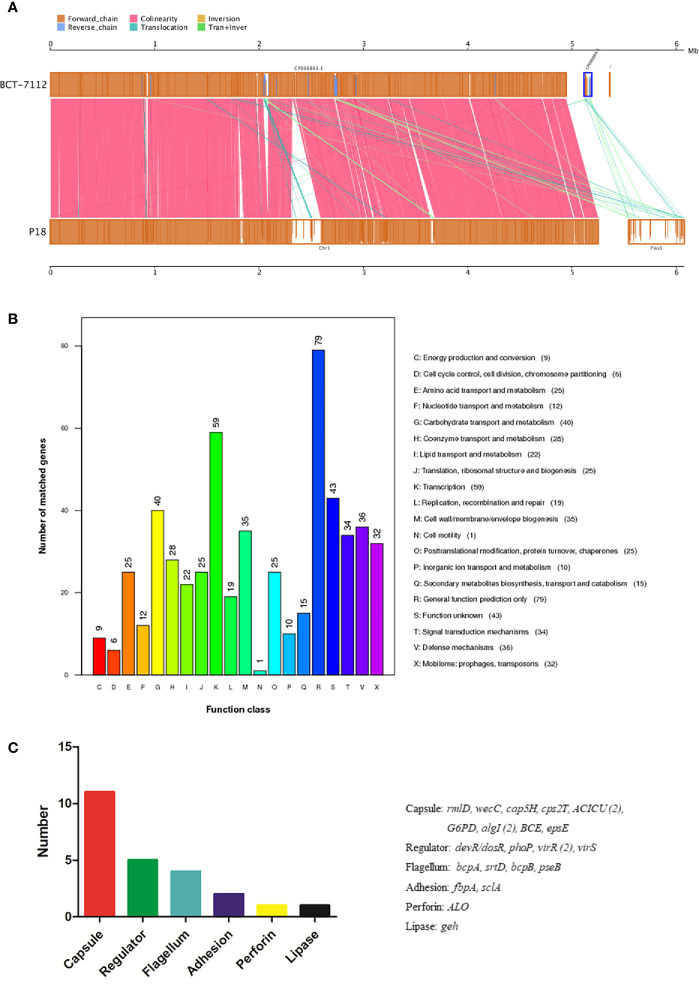
Genome comparison between P18 and BCT-7112^T^. **(A)** Chromosome (left) and plasmid (right) alignment of P18 and BCT-7112^T^. Upper and lower axes of the linear synteny graph are constructed with the same proportion of size reduction of both sequences. Each pair nucleic acid sequence of the two alignments is marked in the coordinate diagram according to its position information, and the height of the filled color in the block indicates similarity of sequence alignment. The color of the lines between the two axes indicates the type of comparison. **(B)** COG functional classification of the P18-specific genes. **(C)** The functional categories of P18-specific virulence genes. The genes of each category are listed on the right.

P18 is similar to *B. toyonensis* BCT-7112^T^ in gene number, average gene length, G+C content, and the number of non-coding RNAs, but has a larger genome size and possesses one, rather than two, plasmids (**Table 1**). However, the size of pBtP18 is much larger than that of either of the plasmids of BCT-7112 ^T^, and is 6.3 times of the combined sizes of the two plasmids of BCT-7112^T^. pBtP18 also possesses more GIs than the BCT-7112^T^ plasmids, which makes the total number of GIs in P18 higher than that in BCT-7112^T^. Most of the chromosomal genes of P18 and BCT-7112^T^ exhibit strong collinearity, but the genes in the plasmids of the two strains exhibit translocation and inversion (**Figure 3A**). P18 and BCT-7112^T^ share 4151 common gene clusters, and contain 1401 and 529 specific genes, respectively. Of the 1,401 P18-unique genes, 960 encode hypothetical proteins with unknown function in the COG database, and 441 were classified into 20 COG functional categories, with the majority of these genes belonging to the categories of R (General function), K (Transcription), G (Carbohydrate transport and metabolism), V (Defense mechanisms), M (Cell wall/membrane/envelope biogenesis), T (Signal transduction mechanisms), and X (Mobilome: prophages, transposons) (**Figure 3B**).

### Virulence-Related Genes of P18

A total of 319 virulence genes were identified in P18 based on a database of known virulence genes (**Supplementary File S1**). Most of these virulence genes are also present in BCT-7112^T^. Twenty-four of the virulence genes are specific to P18 and absent in BCT-7112^T^. These genes are classified into the functions associated with capsule, regulator, flagella, adhesion, perforin, and lipase (**Figure 3C**). P18 carries a number of toxin-related genes, including hemolysins (*hlyIII*, *hblA*, *hblB*, *hblC*, and *hblD*), anthrolysin (*alo*), phospholipase C (SM-PLC, PI-PLC, and PC-PLC), and non-hemolytic enterotoxins *(nheA*, *nheB*, and *nheC*).

### Infectivity and Lethality of P18 to Fish and Mice

**Figure 4:**
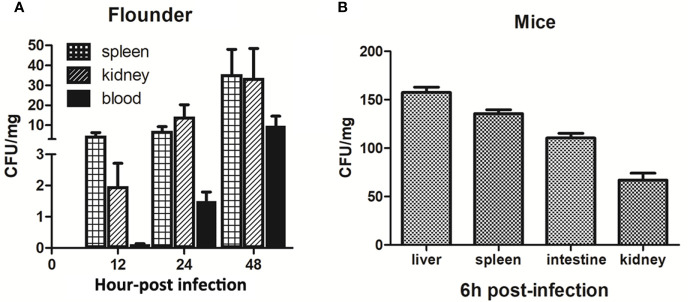
Dissemination of P18 in infected animals. Japanese flounder **(A)** and mice **(B)** were inoculated with P18, and bacterial recovery from the tissues was determined at different time points. The results are the means of triplicate experiments and shown as means ± Standard Error of Mean (SEM).

Live infection study in fish and mice models showed that the LD_50_ of P18 in Japanese flounder and mice were 1.4 × 10^5^ and 2.5 × 10^6^ CFU/g, respectively, when P18 was inoculated into the animals *via* i.m. (for fish) and i.p. (for mice) injection. When inoculated into flounder at the dose of 3 × 10^5^ CFU/g, P18 induced 80% accumulated mortality, whereas *B. subtilis* 168 in no mortality (**Supplementary Figure S2**). P18-injected fish began to exhibit skin ulceration and swelling at 12 hpi, and the symptom worsened over time (**Supplementary Figure S3A**). P18 was detected in the blood, spleen, and kidney of the infected fish from 12 to 48 hpi (**Figure 4A**). Mice infected with 2.5 × 10^6^ CFU/g of P18 exhibited weakness in the limb and vomiting at 4 hpi, and mortality began to occur at 6 hpi (**Supplementary Figure S3B**). At 6 hpi, bacterial dissemination in the liver, spleen, intestine, and kidney of the mice was observed (**Figure 4B**).

### Tissue Lesion Induced by P18

**Figure 5:**
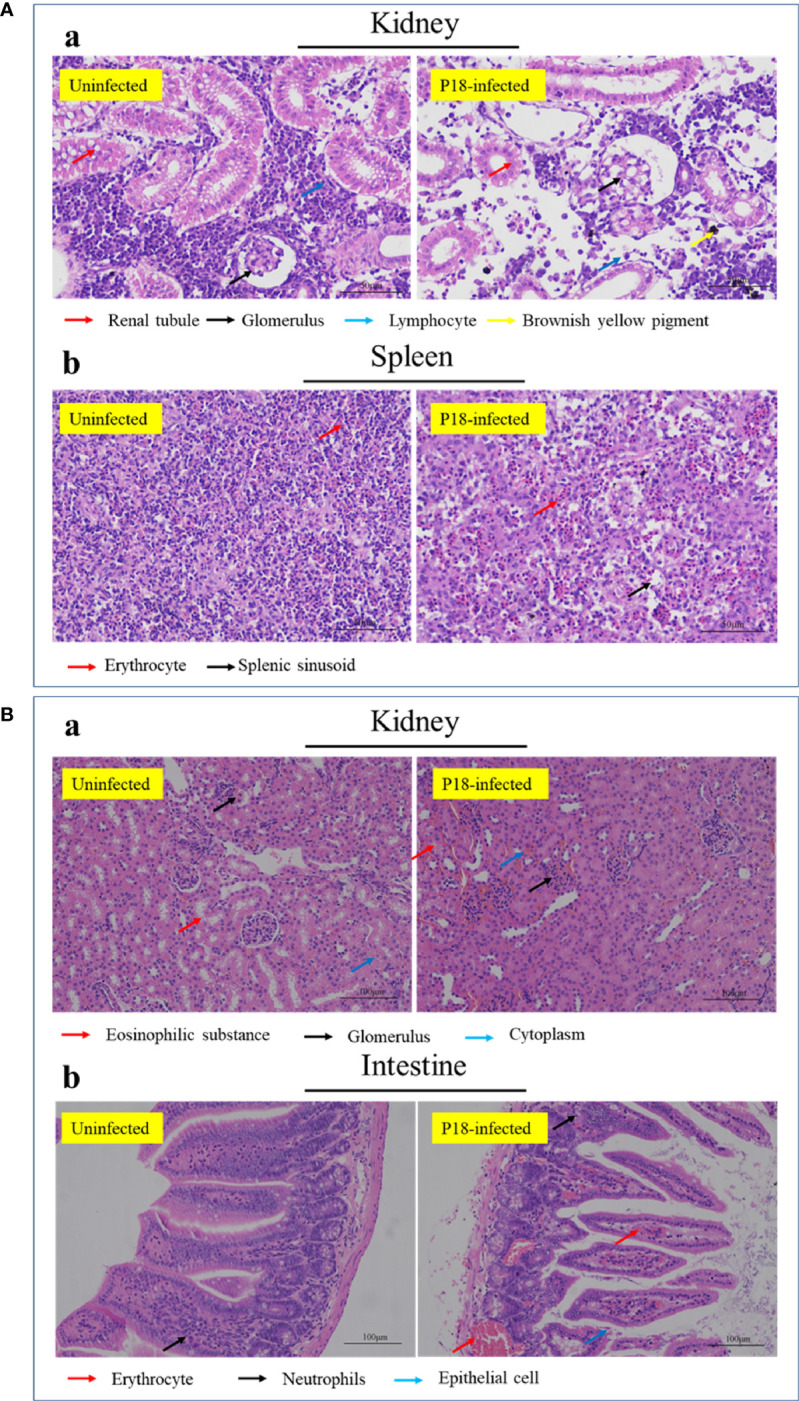
Histopathologic examination of P18-infected fish **(A)** and mice **(B)**. Japanese flounder **(A)** and mice **(B)** were infected with or without P18 for 48 h **(A)** or 6 h **(B)**, and the tissue structures were examined with a microscope. Bar size, 50 **(A)** and 100 **(B)** µm.

Histopathology showed that in P18-infecetd fish, the arrangement of kidney renal tubules became looser, and the lymphocyte number in the mesenchyme dramatically decreased (**Figure 5Aa**). In spleen, there was a considerable reduction in the amount of immune cells in the white pulp and mesenchyme, accompanied by slight expansion of splenic sinus (**Figure 5Ab**). In P18-infecetd mice, kidney exhibited narrowed renal capsules and hemorrhage, and the intestine exhibited abscission of epithelial cells and hyperemia of blood vessels (**Figure 5B**).

### Cytopathic Effect of P18

**Figure 6:**
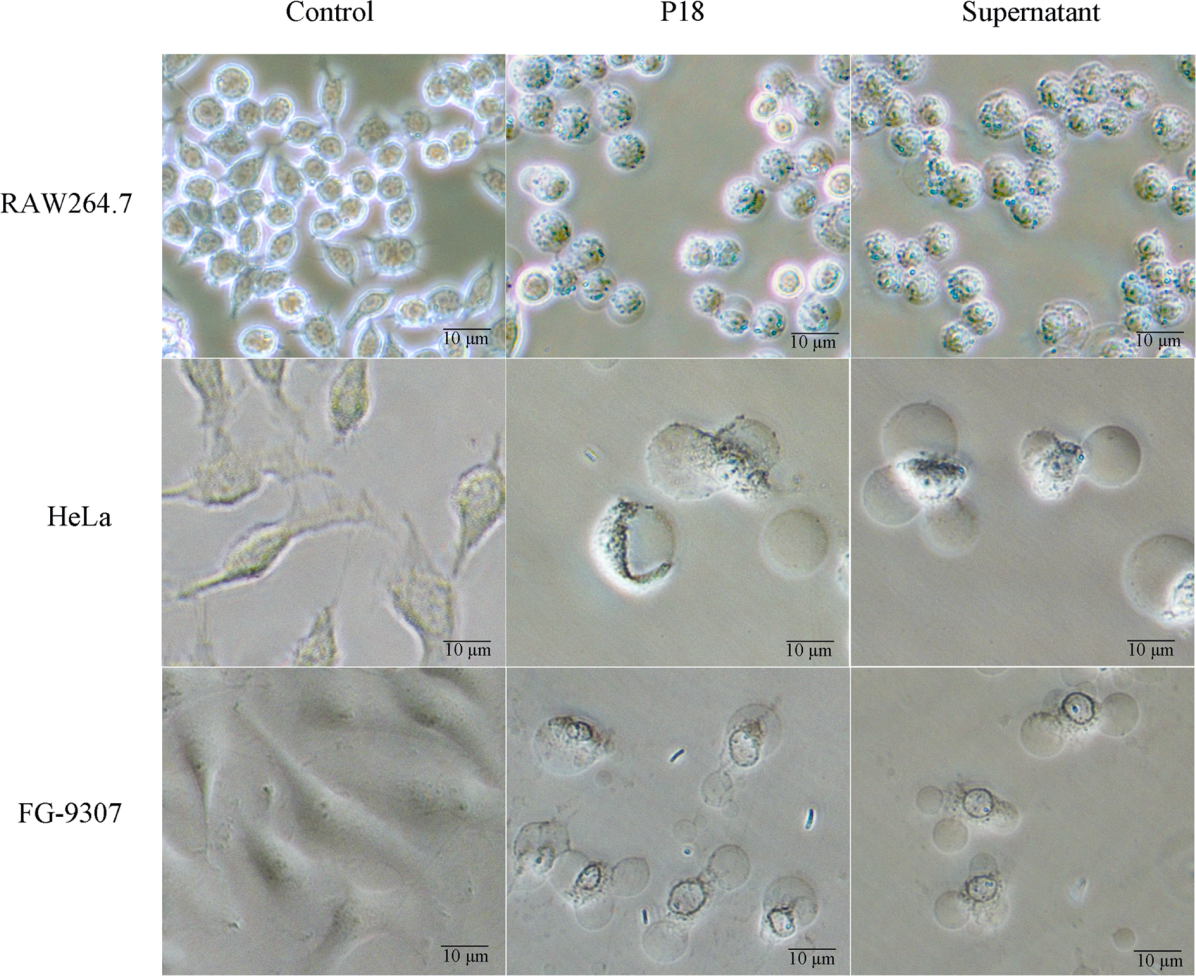
The effect of P18 on mammalian and fish cells. RAW264.7 cells (upper), Hela cells (middle), and FG-9307 cells were treated with or without (control) P18 or its culture supernatant, and the cells were observed with a microscope.

As shown in **Figure 6**, when RAW264.7 cells (mouse monocytes-macrophages) were incubated with P18 at an MOI of 3:1, the cells rapidly rounded out, and cell death was observed at 1.5 h after incubation. When incubated with HeLa cells (human epithelial cells) or the fish epithelial cells FG-9307, P18 induced rapid cellular membrane bubbling and damage that eventually led to cell death. Similar cytopathic effects were observed with the cells treated with the culture supernatant of P18 (**Figure 6**).

### Serum Resistance and Hemolytic Activity of P18

**Figure 7:**
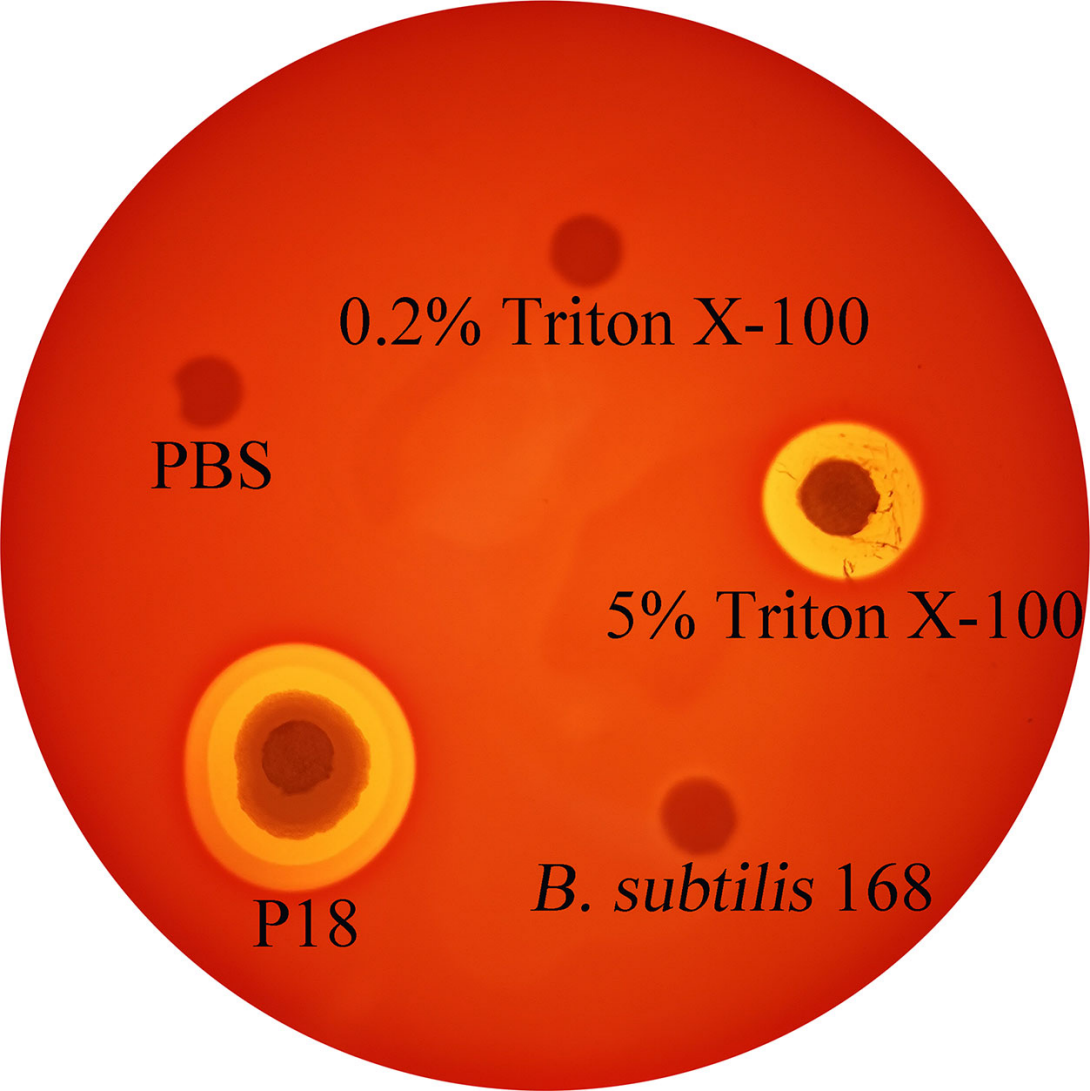
Hemolytic activity of P18. P18, *Bacillus subtilis* 168 (a non-hemolytic bacterium), 5% Triton X-100, 0.2% Triton X-100, and PBS were spotted onto the filter discs in sheep blood agar plate, and hemolysis was observed after 24 h incubation.

Serum resistance analysis showed that P18 exhibited survival rates of 78 and 70% in flounder and mice sera, respectively. Hemolytic analysis indicated that P18 caused lysis of sheep blood cells to the effect comparable to that caused by 5% Triton X-100, but, unlike Triton X-100, P18 induced the formation of double hemolysis halos on the blood agar plate (**Figure 7**).

## Discussion

In this study, we examined the biological features of a *B. toyonensis* strain, P18, isolated from a deep hydrothermal field. We found that P18 was able to form spores and grow at conditions resembling that of the local deep sea environment (low temperature and high salinity). However, we do not know whether P18 exists predominately as vegetative cells or spores or both under the native condition. P18 was classified as a member of *B. toyonensis* based on its high ANI with the type strain of *B. toyonensis*, BCT-7112^T^. BCT-7112^T^ is a soil bacterium isolated in Japan in 1966 for use as a feed additive and was re-classified in 2013 as a novel member of the *Bacillus cereus* group (Jimenez et al., 2013). Compared to BCT-7112^T^, the genome of P18 is 762368 bp larger and consequently possesses 664 more coding genes. One distinguishing feature of P18 is the presence of an exceedingly large plasmid, pBtP18, which in part accounts for the larger genomic size of P18. In contrast to the two plasmids of BCT-7112^T^, which contain a single or no GI, pBtP18 contains 4 GIs with genes involved in, among others, transposition. Consistently, collinearity analysis showed that the genes of pBtP18 exhibit marked translocation and inversion, which is most likely caused by the transposases in the GIs of pBtP18. These observations are in agreement with a previous report which showed that the GIs in the genome of *B. cereus* probably play a role in horizontal gene transfer and, as a result, cause gene gain or loss  (Zhang and Zhang, 2003). Since P18 is a spore-forming bacterium, the possibility exists that it may have originated from a non-deep sea environment, from which the spores were carried to the deep sea. Nevertheless, the distinct genetic features of P18, together with its growth property, observed in our study suggest a living adaption to the deep sea environment.

Although a large amount of genes are shared by P18 and BCT-7112^T^, there are still 1401 P18-unique genes that are absent in BCT-7112^T^. In a previous study of the genomes of 66 deep-sea bacteria, it was found that extracellular peptidase and carbohydrate metabolism genes are widespread in most bacteria (Li et al., 2016). In line with this observation, we found that some of the P18-unique genes with predictable functions are related to carbohydrate transport and metabolism, cell membrane/wall/envelope synthesis, transcription, signal transduction, transposition, and other general functions. However, it is of note that most of the P18-unique genes encode hypothetical proteins that cannot be annotated in function based on the current COG database. It is possible that these hypothetical genes may encode deep sea-unique proteins with functions to facilitate bacterial survival under the specific conditions of the hydrothermal filed.

*B. toyonensis* has been studied primarily as a probiotic for preventing microbial diseases of crops or improving the immune response of animals (Santos et al., 2018). To date, little is known about the pathogenicity of *B. toyonensis*. Several studies have provided evidences that suggested a lack of or low cytotoxic potential for some *B. toyonensis* strains (Guinebretiere et al., 2010; Johler et al., 2018; Abdulmawjood et al., 2019; Carroll et al., 2020; Mendez Acevedo et al., 2020). Two other reports showed that *B. toyonensis* could exhibit larvacidal effect against beet armyworm (Eski et al., 2018) or induce HeLa cell membrane damage (Miller et al., 2018). In our study, a large account of putative virulence genes were identified in P18, including those encoding capsule, flagella, phospholipase C, and cytotoxins, which are known to facilitate the infection of *B. cereus* and other bacteria (Dons et al., 2004; van Asten et al., 2004; Ramarao and Lereclus, 2005). In line with this observation, P18 possesses visible flagella and exhibited apparent swimming and swarming capacities. Live animal infection showed that P18 was able to induce acute infection in fish and mice upon im/ip injection and cause high mortality. Following inoculation, P18 rapidly appeared in multiple internal tissues, and evident histological changes were detected in some organs of the infected animals. These results suggest that P18 possesses *in vivo* invasion capacity that enables the bacteria to disseminate in host tissues and cause pathological lesions.

Genes coding for several types of cytotoxins were found in P18, including *hbl*, *nhe*, *alo*, and *plc*. Hbl and Nhe are prominent cytotoxins of the *B. cereus* group. Hbl can activate the NLRP3 inflammatory response and induce lysis of animal cells such as RAW267.4 (Sastalla et al., 2013). Similarly, Nhe triggers osmotic lysis of the target cells following pore formation in the plasma membrane (Fagerlund et al., 2008). Anthrolysin O (ALO) was identified as a hemolysin and a cholesterol-dependent cytolysin produced by *Bacillus anthracis* and is involved in anthrax pathogenesis (Bourdeau et al., 2009; Tonello and Zornetta, 2012). Phospholipase C is also an inducer of membrane damage by its ability to hydrolyze membrane phospholipids (Goldfine et al., 1993; Goebel and Kuhn, 2000). In accordance with the abundance of cytolytic genes in its genome, P18 displayed strong cytopathic effects on different types of vertebrate cells. The observation of cytotoxicity conferred by the culture supernatant of P18 suggests extracellular toxin production. In addition to fish cells and murine cells, P18 exhibited marked ability to lyse sheep red blood cells. The hemolytic activity of P18 may account for the hemorrhage observed in some of the tissues of P18-infected animals.

In summary, we demonstrated in this study that pathogenic *B. toyonensis* exists in deep hydrothermal systems. We found that *B. toyonensis* P18 possesses a large number of virulence genes and genes of unknown function. Consistently, P18 is highly toxic to fish and mammalian cells and can invade into host tissues and cause mortality. These results provide the first insight into the pathogenicity of *B. toyonensis* associated with deep sea environments.

## Data Availability Statement

The data presented in the study are deposited in the GenBank repository, accession number CP064875 and CP064876.

## Ethics Statement

The animal study was reviewed and approved by Animal Ethics Committee of Institute of Oceanology, Chinese Academy of Sciences.

## Author Contributions

J-cL, HL, and YZ performed the experiments and analyzed the data. JZ obtained the deep sea sample and isolated the bacteria. LS conceived and designed the experiments. J-cL and LS wrote the paper. All authors contributed to the article and approved the submitted version.

## Funding

This work was supported by the grants from Qingdao National Laboratory for Marine Science and Technology (QNLM2016ORP0309), the Strategic Priority Research Program of the Chinese Academy of Sciences (XDA22050402), the Collaborative Research Grant (KLMVI-OP-202002) of CAS Key Laboratory of Molecular Virology & Immunology, Institut Pasteur of Shanghai, Chinese Academy of Sciences, and the Taishan Scholar Program of Shandong Province.

## Conflict of Interest

The authors declare that the research was conducted in the absence of any commercial or financial relationships that could be construed as a potential conflict of interest.
