# Supplementary material for: Characterization of a Deep Sea Bacillus toyonensis Isolate: Genomic and Pathogenic Features
Source: Front Cell Infect Microbiol. 2021 Mar 10;11:629116. doi: 10.3389/fcimb.2021.629116 (PMC7988205; doi:10.3389/fcimb.2021.629116)
Supplement: Supplementary file 2 [file DataSheet_2.doc]

**Characterization of a deep sea *Bacillus toyonensis* isolate: Genomic and pathogenic features**

Jing-chang Luo^a,b,c^, Hao Long^d^, Jian Zhang^e^, Yan Zhao^a,b^, Yong-hua Hu^f^, Li Sun^a,b,c*^

*^a^CAS Key Laboratory of Experimental Marine Biology, Institute of Oceanology, Center for Ocean Mega-Science, Chinese Academy of Sciences, Qingdao, China*

*^b^Laboratory for Marine Biology and Biotechnology, Pilot National Laboratory for Marine Science and Technology (Qingdao), Qingdao, China*

^c^*University of Chinese Academy of Sciences, Beijing, China*

^d^*State Key Laboratory of Marine Resource Utilization in South China Sea, Hainan University, Haikou, Hainan, China*

^e^ *School of Ocean, Yan tai University, Yantai, China*

^*^Correspondence:

Mailing address:

Prof. Li Sun

Institute of Oceanology

Chinese Academy of Sciences

7 Nanhai Road

Qingdao 266071, China

Phone: 86-532-82898829

Email: lsun@qdio.ac.cn

Running title: Characterization of a hydrothermal *B. toyonensis* isolate

**Supplemental data**

**File S1.** Virulence genes of P18 predicted using VFDB (Virulence Factors of Pathogenic Bacteria Database).

Note: This file is in the form of an Excel table and was uploaded separately as “Supplementary File S1”.

**Table S1**. Average Nucleotide Identity (ANI) between P18 and other *Bacillus* strains.

| Strain | Accession number | ANI value (%) |
| --- | --- | --- |
| *Bacillus toyonensis* BCT-7112^T^ | GCA_000496285.1 | 98.67 |
| *Bacillus toyonensis* BV17 | GCA_009799785.1 | 98.59 |
| *Bacillus thuringiensis* ATCC 10792^T^ | GCA_002119445.1 | 91.54 |
| *Bacillus thuringiensis* MYBT18246 | GCA_001685565.1 | 91.43 |
| *Bacillus cereus* ATCC 14579^T^ | GCA_000007825.1 | 91.43 |
| *Bacillus cereus* NC7401 | GCA_000283675.1 | 90.83 |
| *Bacillus mycoides* ATCC 6462^T^ | GCA_000832605.1 | 90.41 |
| *Bacillus mycoides* AH603 | GCA_000161335.1 | 90.24 |
| *Bacillus weihenstephanensis* WSBC 10204^T^ | GCA_000775975.1 | 90.24 |
| *Bacillus wiedmannii strain* FSL W8-0169^T^ | GCA_001583695.1 | 91.22 |
| *Bacillus anthracis* str. Ames | GCA_000007845.1 | 90.80 |
| *Bacillus anthracis* 2002013094 | GCA_000832965.1 | 90.88 |
| *Bacillus pseudomycoides* DSM 12442^T^ | GCA_000161455.1 | 82.49 |

**Figure S1**. Growth analyses of P18 at different pH, temperature, and NaCl conditions (A) P18 was cultured in 2216E medium containing 0-8% (w/v) NaCl. (B) P18 was cultured in 2216E medium with different pH (pH 5–10). (C) P18 was cultured in 2216E medium at different temperatures (16, 28, 37, and 50 °C). (D) P18 was cultured in 2216E agar plate at 4 ℃ for 7 days.


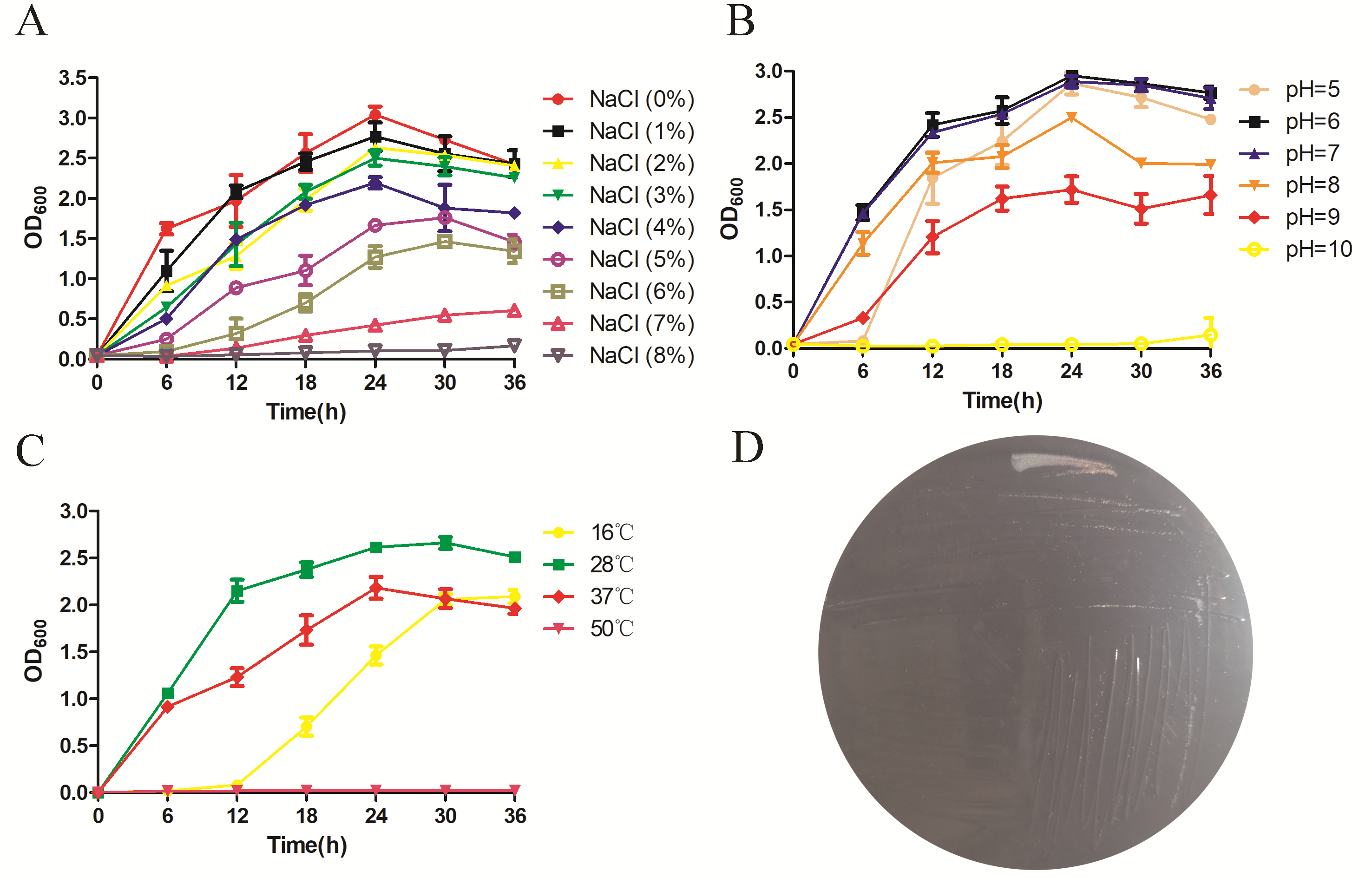


**Figure S2**. Comparison of the mortality-inducing capacity of P18 and *Bacillus subtilis* 168. Japanese flounder were infected with P18 or 168 at the dose of 3×10^5^ CFU/g fish, and the survival of the fish was recorded for 7 days.

**
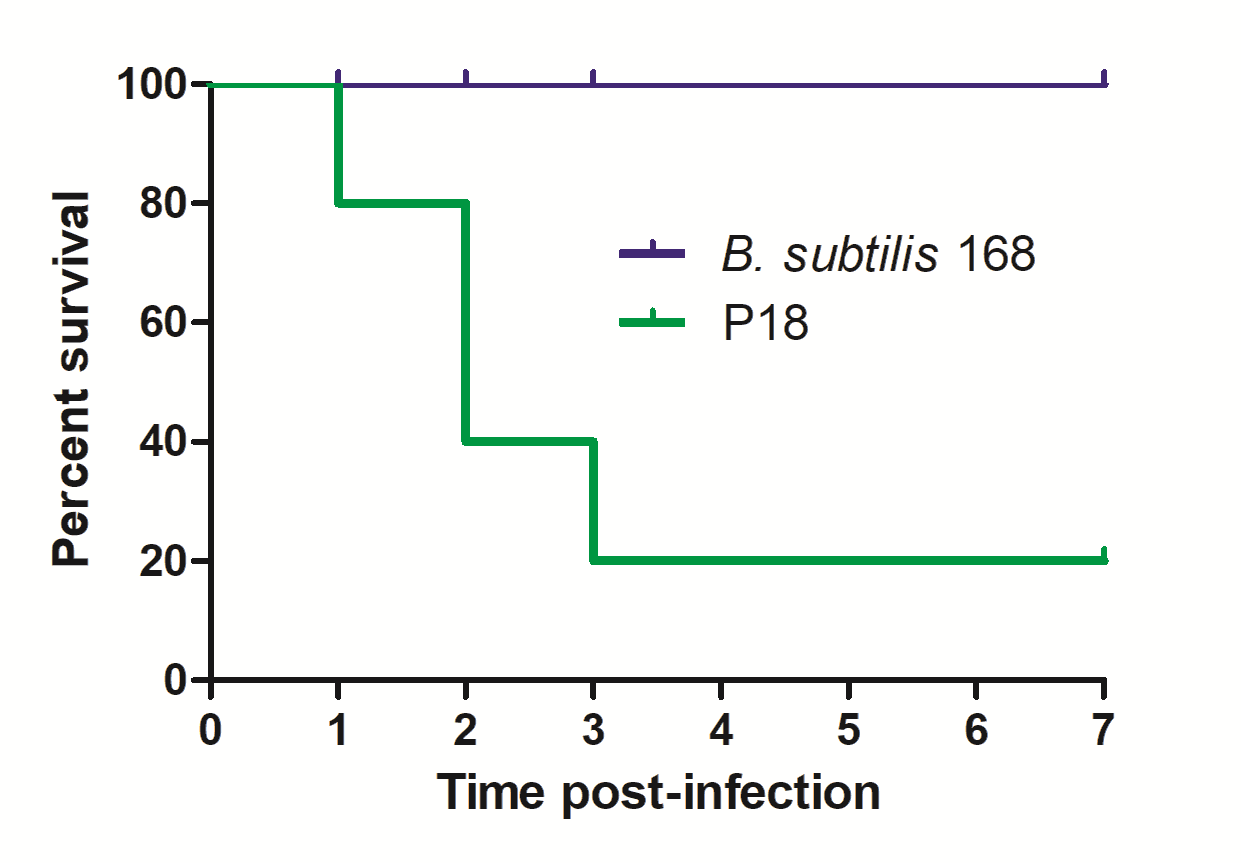
**

**Figure S3**. Clinical symptoms of fish and mice induced by P18. Japanese flounder (A) and mice (B) were infected with or without P18 and observed at different hours post infection (hpi). The blue arrows in (A) indicate the injection sites.

**
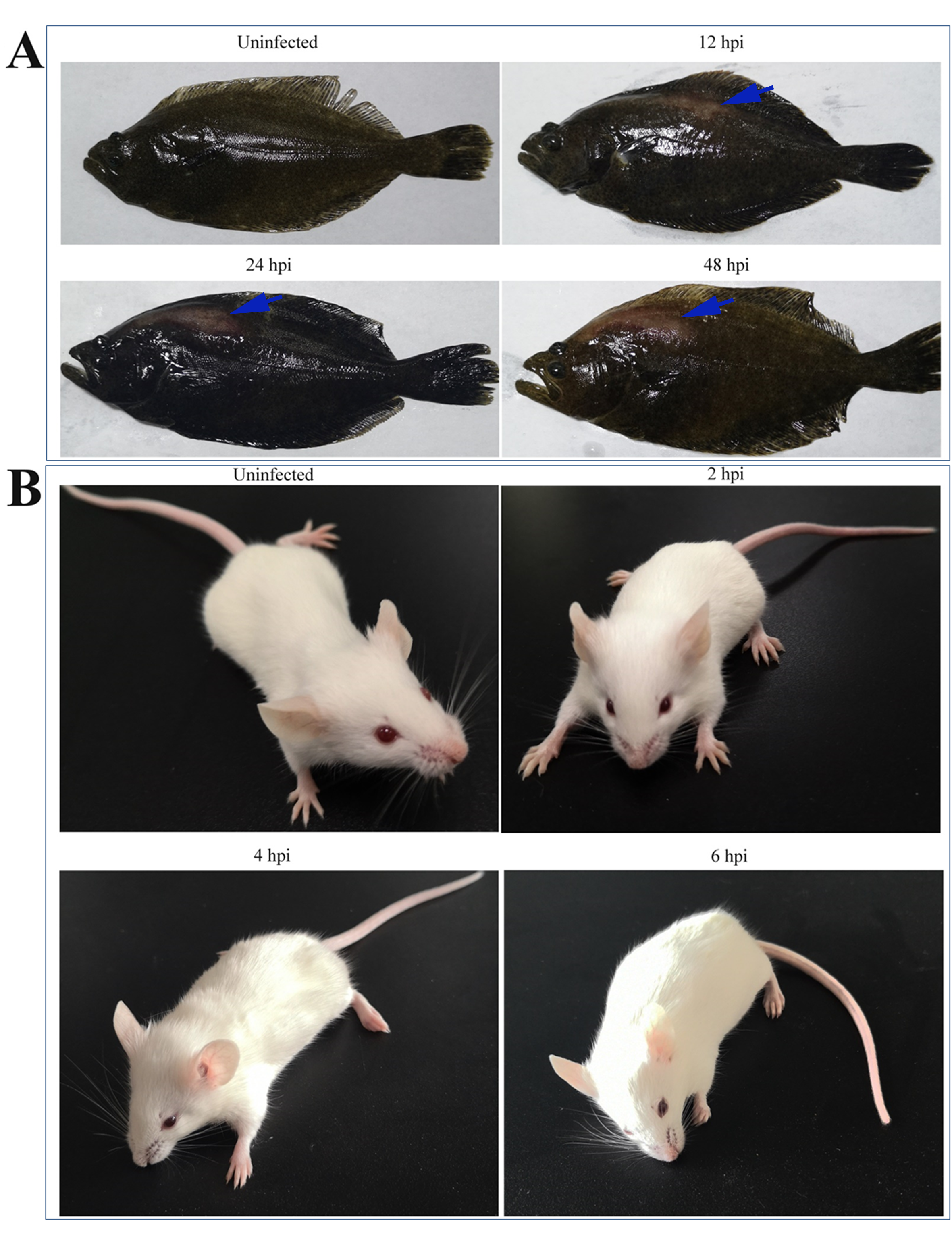
**
